# Immunoproteomic identification of immunodominant antigens independent of the time of infection in *Brucella abortus* 2308-challenged cattle

**DOI:** 10.1186/s13567-015-0147-6

**Published:** 2015-03-01

**Authors:** Jin Ju Lee, Hannah Leah Simborio, Alisha Wehdnesday Bernardo Reyes, Dae Geun Kim, Huynh Tan Hop, Wongi Min, Moon Her, Suk Chan Jung, Han Sang Yoo, Suk Kim

**Affiliations:** Animal and Plant Quarantine Agency, Anyang, Gyeonggi-do 430-757 Republic of Korea; Institute of Animal Medicine, College of Veterinary Medicine, Gyeongsang National University, Jinju, 660-701 Republic of Korea; Department of Infectious Diseases, College of Veterinary Medicine, Seoul National University, Seoul, 151-742 Republic of Korea; Institute of Agriculture and Life Science, Gyeongsang National University, Jinju, 660-701 Republic of Korea

## Abstract

Brucellosis is a vital zoonotic disease caused by *Brucella*, which infects a wide range of animals and humans. Accurate diagnosis and reliable vaccination can control brucellosis in domestic animals. This study examined novel immunogenic proteins that can be used to detect *Brucella abortus* infection or as an effective subcellular vaccine. In an immunoproteomic assay, 55 immunodominant proteins from *B. abortus* 544 were observed using two dimensional electrophoresis (2DE) and immunoblot profiles with antisera from *B. abortus*-infected cattle at the early (week 3), middle (week 7), and late (week 10) periods, after excluding protein spots reacting with antisera from *Yersinia enterocolitica* O:9-infected and non-infected cattle. Twenty-three selected immunodominant proteins whose spots were observed at all three infection periods were identified using MALDI-MS/MS. Most of these proteins identified by immunoblot and mass spectrometry were determined by their subcellular localization and predicted function. We suggest that the detection of prominent immunogenic proteins during the infection period can support the development of advanced diagnostic methods with high specificity and accuracy; subsidiarily, these proteins can provide supporting data to aid in developing novel vaccine candidates.

## Introduction

*Brucella* spp. are the etiological agents for brucellosis, a debilitating and chronic disease infecting a variety of domestic animals and humans. Brucellosis is characterized by abortion and sterility in livestock as well as undulant fever, arthritis and neurological disorders in humans [1]. Definitive diagnosis is commonly performed by isolation and identification of the causative organism(s), but because the isolation is time-consuming and dangerous, serological analysis is widely preferred [2]. Several specific serological tests have been developed for the definitive diagnosis of brucellosis, and these tests have been upgraded repeatedly to obtain reliable data [3]. However, a large number of tests still rely on presumptive evidence of infection. Most serological tests for *Brucella* infection use antibodies against common antigens of *Brucella* [4]. O-polysaccharide (OPS), a well-known immunodominant epitope in smooth lipopolysaccharide (SLPS) is a commonly used antigen in serological tests for the diagnosis of brucellosis [5,6]. Consequently, the serological diagnosis of brucellosis is complicated by cross-reactions of the antibodies against other Gram-negative bacteria, such as *Y. enterocolitica* O:9, which have conserved and highly analogous OPS structures [7,8]. Therefore, it is crucial to discover highly specific *Brucella* antigens that are immunogenic in the host. Several studies have focused on the use of antigenic proteins for alternative diagnostic methods and to improve vaccine efficacy. Recent studies have focused on the use of immunogenic proteins for serodiagnosis of brucellosis [9].

Several immunogenic proteins of *B. abortus* have been identified [10], but the antigens that are immunogenic at different stages of the infection have not been defined. Because *Brucella* causes latent infection, knowledge concerning the different stages of infection is important for the diagnosis and control of the disease. In this study, we obtained antisera against *B. abortus* from experimentally infected cattle at different stages of infection and studied unique immunogenic proteins to validate the immunogenic relationships and potential immunodominant markers at different stages of infection.

## Materials and methods

### Bacterial strains and culture conditions

The standard reference strains *B. abortus* 2308 and *B. abortus* 544, which are known as virulent biovar 1 strain, and *Y. enterocoitica* O:9 used in the present study were obtained from the Laboratory of Bacteriology Division in the Animal and Plant Quarantine Agency, Korea. The bacteria were cultured at 37 °C with aeration until the cells entered stationary phase. Subsequently, the number of viable bacteria was evaluated by plating 10-fold serial dilutions (made using PBS) on Brucella agar plates.

### Preparation of antisera

Twenty-five apparently healthy Korean native heifers (Hanwoo) aged 18–20 months were used in this study. All animals were seronegative for brucellosis before immune challenge, as assessed by the standard tube agglutination test (STAT) and Rose Bengal test (RBT), which are internationally accepted serological tests for bovine brucellosis described by the OIE [5]. The cattle were divided into 3 groups: *B. abortus*-infected (*n* = 10), *Y. enterocoitica* O:9-infected (*n* = 10) and uninfected controls (*n* = 5). Bacterial inoculation was performed as described in previous methods [11]. Briefly, the first group was inoculated with 4 × 10^7^ CFU of *B. abortus* 2308/head injecting a total of 100 μL (50 μL of inoculum per eye) via the intraconjunctival route. The second group was inoculated with 5 × 10^6^ CFU of *Y. enterocoitica* O:9/head 3 times by 1 day interval via subcutaneous injection. The 5 cattle in the uninfected control group were inoculated with sterile PBS. After immune challenge, antisera against *B. abortus* were collected at three stages of infection; early (week 3), middle (week 7), and late (week 10). These time points in three stages of infection were determined based on low serological variations among individuals and high titer values. Samples were collected from all cattle in all groups. Using serological tests, 3 samples of each *B. abortus*-infected antisera (RBT-positive and STAT titers of > 1:400 at 3, 7, and 10 weeks post-challenge), *Y. enterocoitica*-infected antisera (RBT-negative and STAT titers of 1:200 against *Brucella* antigen at 3, 7 and 10 weeks post challenge), and non-infected sera were selected and used for immune analysis. The experimental procedures were approved by the ethical committee as NVRQS-AEC-2008-12, and the infected animals were euthanized according to the protocol of the Institution for Animal Care & Use Committee in Korea.

### Preparations of antigens

Antigens were prepared as a protein mixture of whole cells including cell envelopes for proteomic analysis using a modification of a previously described procedure [10]*.* Briefly, *B. abortus* 544 cultures were centrifuged at 8000 × *g* for 20 min at 4 °C and washed 3 times with ice-cold PBS (pH 7.6) by centrifugation. The bacterial pellet was resuspended in 50 mM Tris–HCl (pH 7.6) containing a complete protease inhibitor cocktail (PIC) and then sonicated on ice using a Sonifier 750 (Branson Ultrasonics, USA). The sonicated solution was centrifuged at 12 000 × *g* for 1 h at 4 °C, and the pellet was resuspended in lysis buffer (5 M urea, 2 M thiourea, 2% CHAPS, 1% SB 3–10, 1% DTT, and containing PIC) followed by incubation at 22 °C for 1 h with vigorous agitation. After centrifugation at 100 000 × *g* for 30 min, the supernatant was collected. The protein concentration was quantified using the Bradford assay [12].

### Isoelectric focusing (IEF) and 2D SDS-PAGE

IEF and 2DE were conducted using a previously described method [13] with modifications. Eighteen-centimeter IPG strips (pH 3–10 and 4–7, GE Healthcare, USA) were rehydrated for 14 h at 22 °C with the lysed proteins and rehydration buffer (7 M urea, 2 M thiourea, 2% CHAPS, 0.2% DTT, 0.5% IPG buffer (pH 4–7), and 0.002% bromophenol blue). IEF was performed on a Protean IEF gel (GE Healthcare) at 20 °C for 14 h using the following conditions: 500 V for 1 h, gradient phase of 1000 V for 1 h, 1000 V for 3 h, gradient phase of 10 000 V for 3 h, 10 000 V for 5 h, 50 V for 30 min and a final phase of 50 V for 30 min. After IEF, each strip was equilibrated in 5 mL of equilibration buffer I (6 M urea, 50 mM Tris–HCl pH 8.8, 1% DTT, 30% glycerol, 2% SDS, and 0.002% bromophenol blue) for 15 min at 22 °C and then in equilibration buffer II (6 M urea, 50 mM Tris–HCl pH 8.8, 2.5% iodoacetamide, 30% glycerol, 2% SDS, and 0.002% bromophenol blue) under the same conditions. The equilibrated strips were loaded on the top of 12% SDS-polyacrylamide gels and sealed with melted 1% agarose solution. The proteins were two-dimensionally separated in resolving buffer (25 mM Tris pH 8.8, 192 mM glycine, 0.1% SDS) using Criterion electrophoresis equipment (Bio-Rad) equipped with a cooling device (Lauda E100, Germany), kept at 25 °C and supplied with regular power in two steps: 5 W/gel for 30 min and 20 W/gel until the protein dye reached the bottom of the gel (approximately 5 h). The separated proteins were transferred to PVDF membranes (Millipore, USA) for immunoblotting analysis; simultaneously, replicate gels containing the same protein samples were silver-stained to visualize the proteins. Three replicates of 2DE were performed in independent experiments.

### Immunoblotting with antisera

The proteins were completely transferred to membranes using the TE70/77 PWR Semi-Dry Transfer Unit (GE Healthcare) according to the manufacturer’s instructions. The membranes were blocked for 1 h at room temperature using 5% rabbit serum in Tris-buffered saline containing 0.1% Tween-20 (TBS-T) and then washed three times for 20 min in TBS-T. The blots were incubated overnight at 4 °C with a 1:500 dilution of the antisera derived from the immune-challenged and control cattle. The blots were then incubated for 1 h at room temperature with a 1:5000 dilution of goat anti-bovine IgG HRP-conjugated antibody (Sigma, USA). After washing, the immunolabeling was detected using ECL Western blotting reagents (GE Healthcare). Finally, specific immunogenic proteins were visualized using a ChemiDoc XRS Camera and the Quantity One 1D analysis software (Bio-Rad).

### Gel image analysis and in-gel trypsin digestion

The silver-stained 2D gels were scanned using an ImageScanner™ (GE Healthcare) and cropped using ImageQuant TL (GE Healthcare). Automatic gel-image alignment and spot detection along with spot matching were performed using Progenesis SameSpots v 2.0 (Nonlinear Dynamics) to allow for more accurate spot identification [14]. Each gel was run in triplicate in parallel with three independent sample preparations. The spot matching across all gels without omitting values was set as a requirement for spot merging for data analysis. An average gel with best resolution was generated using the three independent replicates by including only those protein spots that were present in at least two of the replicates. The common spots, in keeping with shape and intensity over all replicates, were selected for normalization of spot volumes to equalize the probable variation in staining trait. The gel containing all spots on final average gel was used and transferred to the PVDF membrane which subsequently was subjected to react with antisera from cattle. In addition, image alignment and spot matching analyses were performed on the gel spots and the immunogenic protein spots detected by immunoblotting. The selected spots were manually excised from the gels, and the gel plugs containing the proteins were enzymatically digested with porcine trypsin (modified sequencing grade; Promega, USA) as described previously [13]. The spots were incubated with 50 mM ammonium bicarbonate (NH_4_CO_3_, pH 7.8)/50% acetonitrile (ACN) for 1 h at 22 °C to de-stain them and were washed and then dehydrated in ACN. The dehydrated spots were vacuum-dried to remove the solvent and then rehydrated overnight at 37 °C by digestion with trypsin (10 ng/μL) in 50 mM NH_4_CO_3_ (pH 7.8). The tryptic peptides were extracted with 0.1% trifluoroacetic acid (TFA)/50% can, and the combined extracts were vacuum-dried by centrifugation and resuspended in 0.5% TFA. The peptide mixture was desalted using ZipTip plates (Millipore) and then eluted with 0.2% TFA/50% ACN. Finally, the resulting solution was mixed with the matrix (10 mg/mL α-cyano-4-hydroxycinnamic acid in 50% ACN/1% TFA).

### Protein identification by MALDI-TOF MS /MS analysis

All spectra were collected using an ABI 4700 proteomics analyzer Plus TOF-TOF Mass Spectrometer (Applied Biosystems, USA). MS/MS data were obtained using this instrument with a Nd:YAG laser with a 200 Hz repetition rate, and accumulation of up to 4000 shots were performed for each spectrum from which the three highest intense peaks were processed to an enhanced resolution. When the three intense peaks were subjected to downstream analysis, these were ignored for a period of 60 s. MS/MS mode was operated with 2 keV collision energy supplying air as the collision gas, which resulted in completion of nominally single collision conditions. MS/MS data were obtained using the default instrument calibration without internal or external calibration. The quality control parameters included based on the Mascot algorithm were the following: maximum of one missed cleavage permitted by trypsin, fixed modification (including residue specificity) of carbamidomethyl, variable modifications (including residue specificity) of oxidation, charge state of 12 to 14, mass tolerance for peptide ion (m/z) of 0.1 to 0.2 Da, cut-off score/expectation value for accepting individual MS spectra of highest expectation (probability on profound search, PPS). All protein identifications were made by only single protein spot and were collected using a score with the minimal number of high quality peptides per protein is 22. Peptide mass data were used to query the NCBI protein sequence and annotated genome databases of *Brucella* using the Mascot search engine (Matrix Science, London, UK) [15]. Based on the sequences identified using mass spectrometry, biological information on the chosen proteins was retrieved using the EXPASY database [16]. The sub-cellular localizations of bacterial proteins were predicted using PSORTb v. 2.0.4 [17]. Functional annotations were made based on the cluster of orthologous groups (COG) protein database generated by comparing all of the complete sequences of microbial genomes from the NCBI COG [18].

## Results

### 2DE profiles of whole-cell antigens from *B. abortus* 544

**Figure 1:**
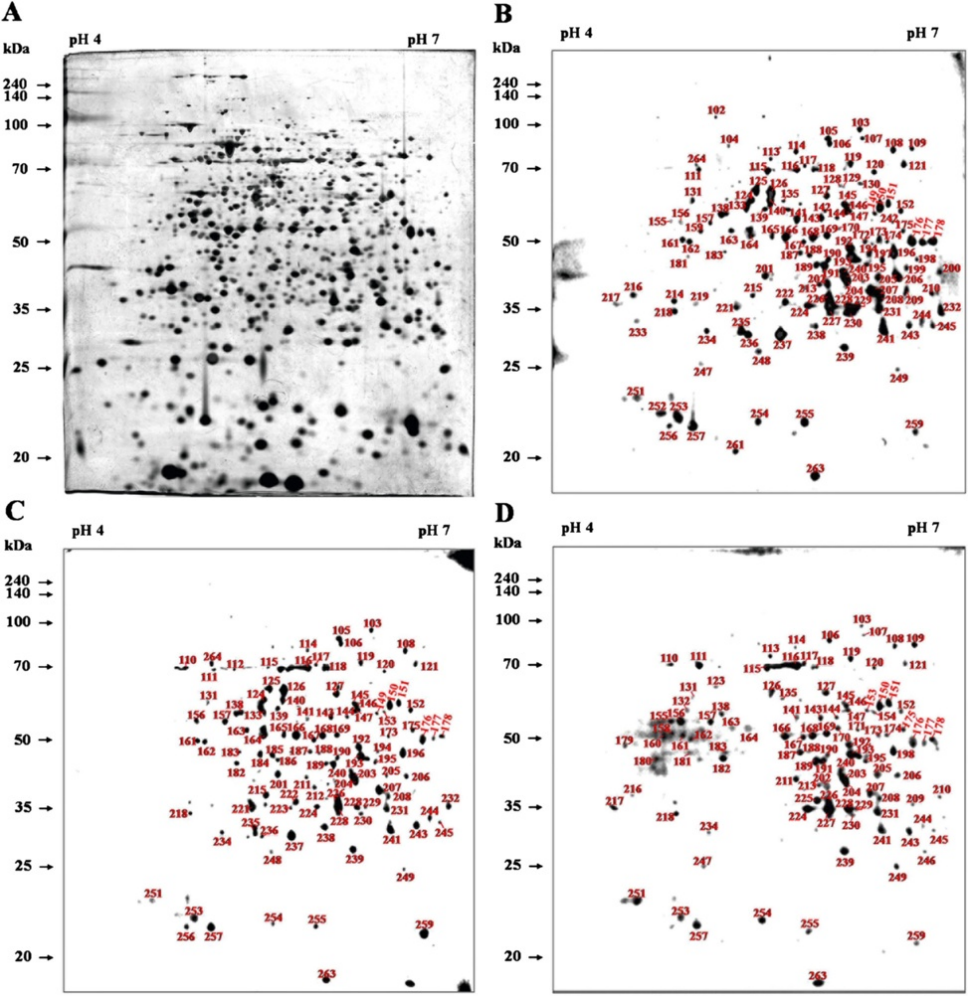
**2DE profile of** ***B. abortus*** **proteins and immunoblotting with antisera from** ***B. abortus*** **-infected cattle. (A)** 2DE profile of proteins from *B. abortus* detected on silver-stained 2DE gels within the p*I* range 4–7. Immunoblotting analyses were performed with antisera from cattle after 3 **(B)**, 7 **(C)**, and 10 weeks **(D)** of challenge with *B. abortus*. Three replicates of 2DE analysis were performed in the independent experiments.

The annotated 2DE proteome map of whole-cell proteins from *B. abortus* 544 is shown in Figure 1A. A total of 1181 protein spots were detected on the silver-stained 2DE gels within the p*I* and molecular weight (*M*_*r*_) ranges of 4–7 and 20–240 kDa, respectively. The 2DE map profiles of the best resolution were obtained from most of the detected spots in the 2DE gels of the three replicates from separate experiments. These replicates were selected based on equal 2D patterns and spot numbers reactive to individual serum from three different infection periods. The mean p*I* and *M*_r_ of all protein spots detected were 5.62 and 40.52 kDa, respectively. Using the broad pH range from 3–10, the protein spots with p*I* < 4 or p*I* > 8 were detected at relatively low resolution with few protein spots.

### Immunogenic proteins in *B. abortus* at different infection periods and comparison with cross-reacting bacteria

**Figure 2:**
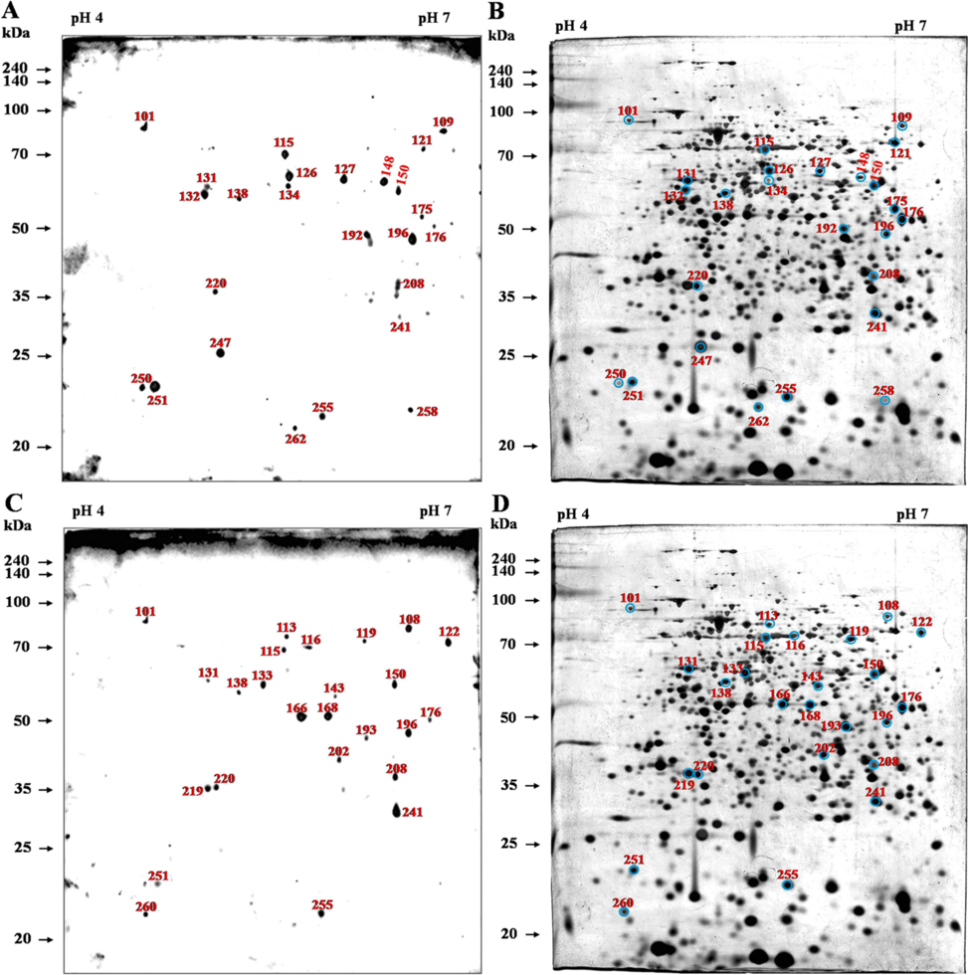
**2DE analysis and the immunoblotting profile detected using sera from non-infected and** ***Y. enterocolitica*** **-infected cattle.** A total of 25 immunoreactive dots were observed using the non-infected **(A)** and *Y. enterocolitica*-challenged **(C)** bovine sera, and the corresponding proteins are labeled on the 2DE gel [NC **(B)** and YP **(D)**]. The numbers represent the serial numbers of the immunoreactive proteins in immunoblotting analyses.

**Figure 3:**
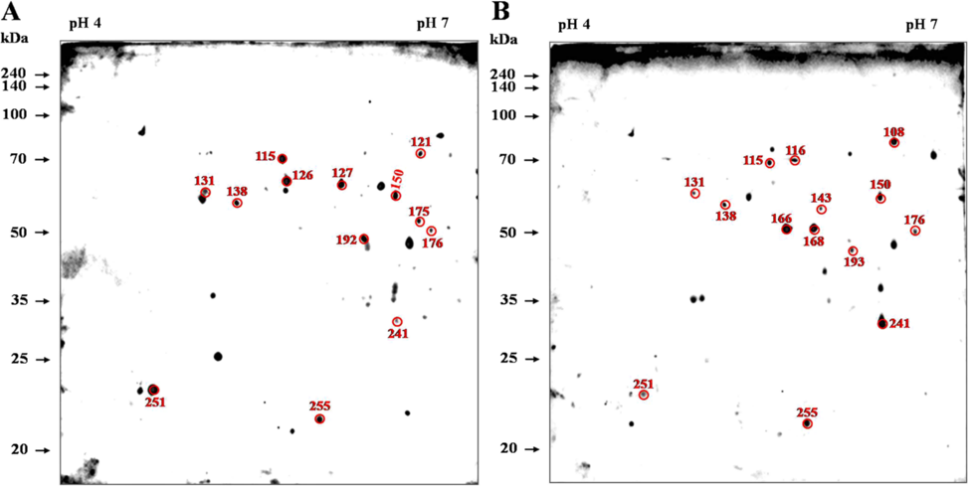
**Comparative 2DE analysis of** ***B. abortus*** **proteins and immunoblotting profile of non-specific reactions.** A total of 13 immunoreactive spots of common antigens that responded to the negative sera from non-infected cattle **(A)** and positive sera of *Y. enterocolitica* **(B)**, and three types of sera from cattle after 3, 7 and 10 weeks of challenge with *B. abortus* were selected. The numbers represent the serial numbers of the immunoreactive proteins in immunoblot analyses.

**Figure 4:**
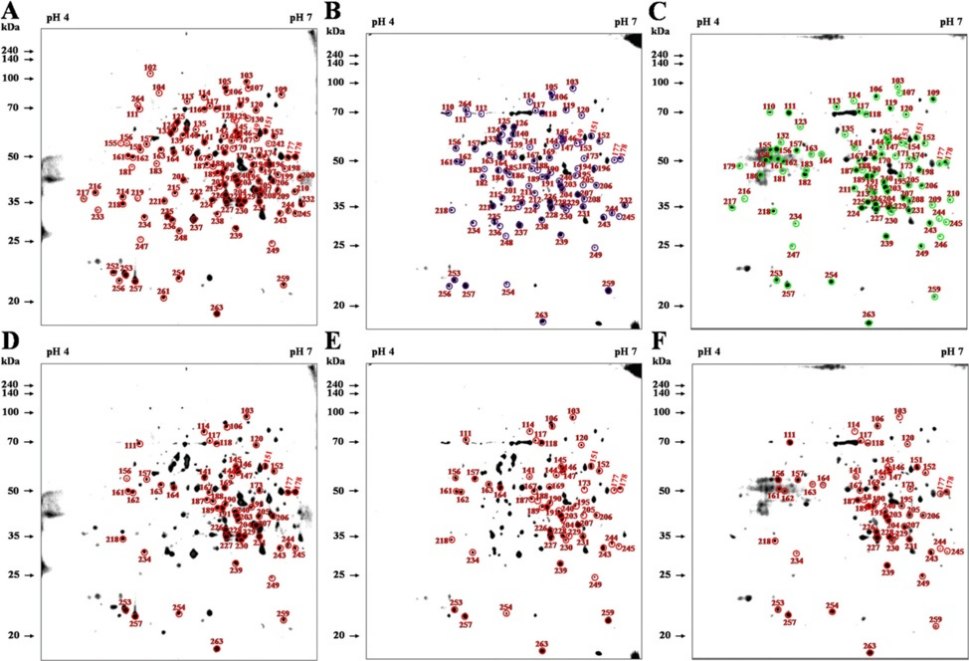
**Immunoblotting profile of** ***B. abortus*** **proteins responded with** ***B. abortus*** **-infected bovine antisera excluding non-specific proteins.** Immunoblotting analyses were performed with antisera from cattle after 3, 7, and 10 weeks of challenge with *B. abortus*. After excluding non-specific reactions, a total of 101 **(A)**, 84 **(B)**, and 78 **(C)** immunoreactive dots, as well as 55 protein spots that reacted with antisera at all 3 stages **(D** **, E, and** **F)**, were selected and labeled. The numbers represent the serial numbers of the immunoreactive proteins in immunoblot analyses.

**Figure 5:**
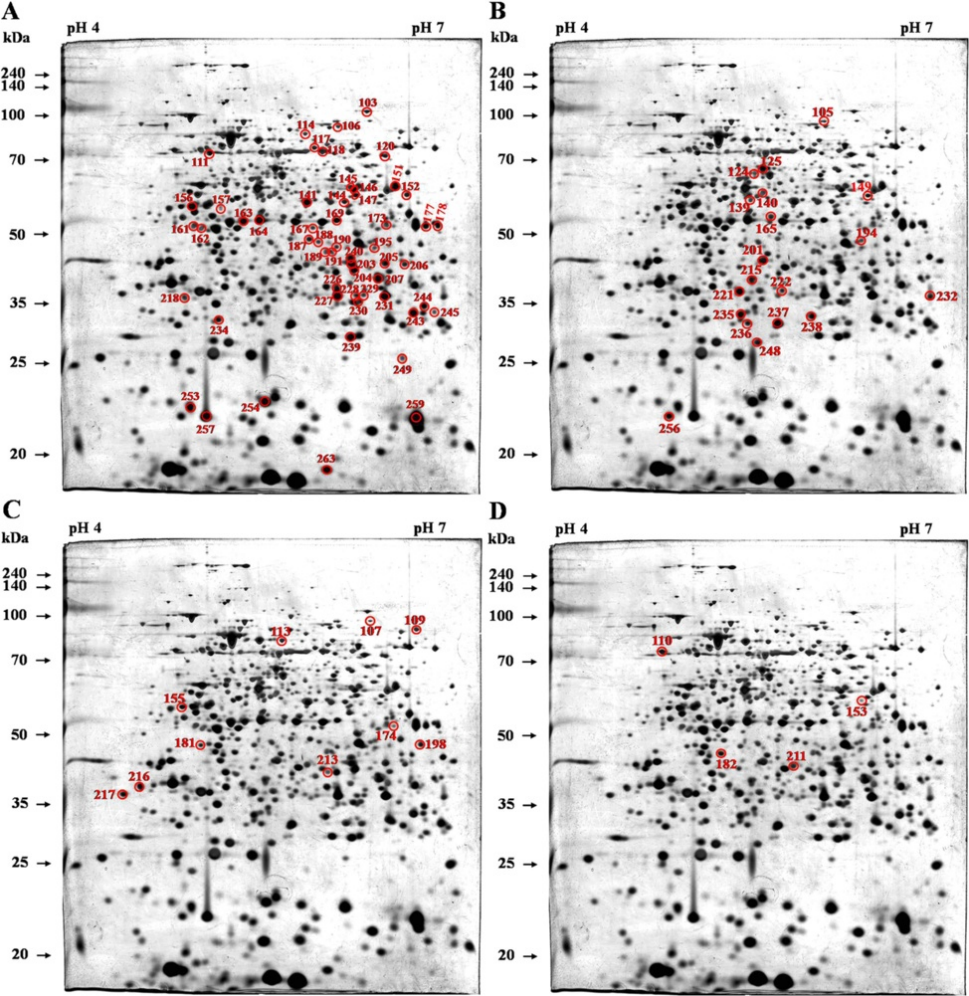
**Comparative 2DE analysis of** ***B. abortus*** **proteins and immunoblotting profiles of specific reactions. (A)** A total of 55 immunoreactive spots of antigen that responded to antisera from cattle after 3, 7 and 10 weeks of challenge with *B. abortus* were selected. A total of 19, 10, and 4 immunoreactive spots of antigen responded to antisera at 2 time-points: **(B)** after 3 and 7 weeks of challenge, **(C)** after 3 and 10 weeks, and **(D)** after 7 and 10 weeks; these spots were selected and labeled on the 2DE gel. The numbers represent the serial numbers of the immunoreactive proteins in immunoblot analyses.

By immunoblotting the diverse *B. abortus* proteins detected on 2DE gels, 134, 110, and 106 proteins were recognized using positive antisera from *B. abortus-*infected cattle at 3, 7, and 10 weeks of infection, respectively (Figures 1B-D). The negative sera from the non-infected cattle and the positive antisera from the cattle infected with *Y. enterocolitica* O:9 were also used for immunoblotting to exclude non-specific or cross reactions. Few reactions (25 protein spots) were observed using the negative control (NC) and *Y. enterocolitica* O:9-positive (YP) antisera (Table 1 and Figure 2). The spots reacting to the *B. abortus*-positive (BP) antisera that overlapped with those reacting to the NC (13 spots) and YP (13 spots) antisera were excluded (Figure 3). Among the immunogenic proteins that were not from non-specific and cross-reacting spots, 120 immunodominant proteins (Table 1) were observed using the antisera collected during at least one of the three infection periods, whereas 101, 84, and 78 proteins were specifically observed using BP antisera collected at weeks 3, 7, and 10, respectively (Table 2 and Figure 4). Fifty-five common antigens were predominantly specific to the BP antisera at all three stages of infection (Figure 5A). The percent similarity, calculated as the number of proteins that reacted to the antisera, was 45.83%, suggesting that highly immunogenic proteins were present in the bovine serum within 10 weeks of infection (Table 1). In addition, 19, 10, and 4 common immunoreactive spots were observed at 3 and 7 (Figure 5B), 3 and 10 (Figure 5C), and 7 and 10 weeks (Figure 5D), respectively. Furthermore, 17, 6, and 9 non-matched immunoreactive protein spots were observed, and the percent independence of these immunoreactions were 16.84%, 7.14%, and 11.54% at 3, 7, and 10 weeks, respectively (Table 2).

**Table 1:** **Comparison of immunoreactive proteins of** ***B. abortus*** **after immune challenge in cattle**

**Antisera immunoreactions compared**					**No. of matched protein spots**	**Total no. of protein spots**	**Similarity (%)** ^**a**^
**BP**			**NC**	**YP**			
**Week 3**	**Week 7**	**Week 10**					
+^b^	+	+	+	+	8	162	4.94
+	+	+	+	-	5	137	3.65
+	+	+	-	+	5	137	3.65
+	+	+	-	-	55	120	45.83
+	+	-	-	-	19	42	45.24
+	-	+	-	-	10	36	27.78
-^c^	+	+	-	-	4	19	21.05

NC - negative control, YP - *Y. enterocolitica*-positive sera, BP - *B. abortus*-positive sera.

^a^The percent similarity was calculated as the number of proteins common to the compared antisera immunoreactions divided by the total number of proteins in these antisera immunoreactions × 100.

^b^Positive reaction detected in immunoblotting.

^c^Negative reaction detected in immunoblotting.

**Table 2:** **Comparison of immunoreactive proteins of** ***B. abortus*** **that reacted independently with BP**

	**Antisera (BP) immunoreactions compared**	**No. of non-matched protein spots**	**Total no. of protein spots**	**Independence (%)** ^**a**^
Periods of challenge	week 3	17	101	16.84
	week 7	6	84	7.14
	week 10	9	78	11.54

BP - *B. abortus*-positive sera.

^a^The percent independence was calculated as the number of non-matched proteins to the antisera immunoreactions compared with others divided by the total number of proteins in these antisera immunoreactions × 100.

### Identification of immunogenic proteins at different infection stages

Amongst the 55 immunogenic proteins detected at all three stages of infection, the signal intensities of 23 immunogens were higher than the average when normalized to the total valid spot intensity; these 23 proteins were analyzed using MALDI- MS/MS. The data revealed that several novel immunogenic proteins with diverse ORF had varying values for *M*_r_ and p*I* in MALDI-MS/MS (Table 3). NCBI BLAST searches of the proteins identified using MALDI-MS/MS show that 10 (43.5%), 2 (8.7%) and 2 (8.7%) proteins were predicted to have cytoplasmic, outer membrane-bound periplasmic and ribosomal localization, respectively. However, 9 spots (39.1%) were unknown proteins. Analysis of each identified protein indicates that 13 of the 23 proteins participate in multiple enzymatic activities. Notably, three hypothetical proteins (spots 187, 218 and 257) encoded by different ORF had putative molecular functions such as catalytic and protein disulfide oxidoreductase activities. The experimental p*I* and *M*_r_ values from MALDI-MS/MS identification were consistent with the theoretical values for most identified proteins, with the exception of spot 146, which was identified as an ABC transporter substrate-binding protein; the experimental and theoretical *M*_r_ values for this spot had the highest deviations (6.4).

**Table 3:** **Identification of matched immunoreactive proteins of** ***B. abortus*** **that reacted with** ***B. abortus*** **-positive bovine antisera**

**Spot no.**	**Gene name**	**Gene ID** ^**a**^	**Protein identification**	**Protein ID** ^**a**^	**Accession no** ^**a**^	**Sequence length**	**Locus tag** ^**a**^	**Score**	***M***_***r***_		**p** ***I***	**Sequence coverage (%)**	**Subcellular location** ^**c**^	**COG functional category** ^**d**^
									**Experimental**	**Theorectical** ^**b**^				
118	BruAb2_0325	3341905	aldehyde dehydrogenase	YP_223118.1	Q579C7	500	BruAb2_0325	249	53744	53435	5.64	29	unknown	G: Carbohydrate transport and metabolism
146	BruAb2_0024	3341776	branched chain amino-acid ABC transporter substrate-binding protein	YP_222837.1	Q57A58	471	BruAb2_0024	438	50740	44322	6.43	25	periplasmic space	E: Amino acid transport and metabolism
151	aspC	3339882	aspartate aminotransferase	YP_222177.1	Q57C18	400	BruAb1_1488	193	43812	43554	5.94	18	unknown	F: Nucleotide transport and metabolism
161	BruAb1_0775	3339474	3-hydroxyisobutyryl-CoA hydrolase	YP_221504.1	Q57DZ1	349	BruAb1_0775	258	38120	37802	4.84	27	unknown	H: Coenzyme transport and metabolism
162	tsf	3340671	elongation factor Ts	YP_221867.1	Q57CX8	305	BruAb1_1167	367	32061	31491	5.03	35	cytoplasm	J: Translation, ribosomal structure and biogenesis
164	mdh	3340925	malate dehydrogenase	YP_222574.1	Q57AX1	320	BruAb1_1903	76	33854	33704	5.24	7	cytoplasm	G: Carbohydrate transport and metabolism
169	tbpA	3340057	thiamine transporter substrate binding subunit	YP_222421.1	Q57BC4	334	BruAb1_1742	310	36843	36752	6.06	19	periplasmic space	H: Coenzyme transport and metabolism
178	BruAb1_1058	3341091	cysteine synthase A	YP_221767.1	Q57D78	342	BruAb1_1058	417	34445	36701	5.94	30	cytoplasm	E: Amino acid transport and metabolism
187	BruAb2_0291	3341871	hypothetical protein BruAb2_0291	YP_223086.1	Q579F9	330	BruAb2_0291	267	35457	35251	5.50	26	unknown	R: General function prediction only
203	rpsB	3340672	30S ribosomal protein S2	YP_221868.1	Q57CX7	256	BruAb1_1168	259	29308	27999	5.88	29	ribosome	J: Translation, ribosomal structure and biogenesis
204	ubiG	3340925	3-demethylubiquinone-9 3-methyltransferase	YP_415219.1	Q2YLN5	248	BAB1_1875	410	27653	27486	5.79	31	cytoplasm	E: Amino acid transport and metabolism
207	dapB	3341712	dihydrodipicolinate reductase	YP_223731.1	Q576R4	268	BruAb2_0991	536	28792	27605	5.92	45	cytoplasm	E: Amino acid transport and metabolism
218	BruAb2_0647	3342272	hypothetical protein BruAb2_0647	YP_223419.1	Q577X6	224	BruAb2_0647	302	24961	24805	4.83	34	unknown	Q: Secondary metabolites biosynthesis, transport, and catabolism
227	BruAb2_0628	3342294	metal-dependent hydrolase	YP_223400.1	Q577Z5	237	BruAb2_0628	511	25223	25124	5.58	51	cytoplasm	R: General function prediction only
228	msrA	3341844	methionine sulfoxide reductase A	YP_223747.1	Q576P8	218	BruAb2_1009	167	24230	24017	5.65	20	cytoplasm	O: Posttranslational modification, protein turnover, chaperones
231	BruAb1_1470	3340810	50S ribosomal protein L25	YP_222213.1	Q57BY2	207	BruAb1_1470	171	22369	22383	5.91	47	ribosome	J: Translation, ribosomal structure and biogenesis
239	BruAb1_0588	3339410	Fe-Mn superoxide dismutase	YP_221327.1	Q57EG8	199	BruAb1_0588	359	22526	22540	5.83	37	unknown	P: Inorganic ion transport and metabolism
240	rocF	3341875	arginase	YP_223125.1	P0A2Y1	306	BruAb2_0333	188	33415	33182	5.63	24	unknown	P: Inorganic ion transport and metabolism
243	gpm	3341713	phosphoglyceromutase	YP_223732.1	Q576R3	206	BruAb2_0992	446	22929	22886	6.16	43	cytoplasm	G: Carbohydrate transport and metabolism
253	secB	3339678	preprotein translocase subunit SecB	YP_222709.1	P0C125	163	BruAb1_2047	343	17924	17878	4.89	46	cytoplasm	O: Posttranslational modification, protein turnover, chaperones
254	ndk	3339959	nucleoside diphosphate kinase	YP_221449.1	Q57E46	140	BruAb1_0713	58	15269	15278	5.27	20	cytoplasm	F: Nucleotide transport and metabolism
257	BruAb2_0845	3341366	hypothetical protein BruAb2_0845	YP_223598.1	Q577E7	177	BruAb2_0845	500	18506	18517	5.02	43	unknown	S: Function unknown
263	ohr	3341640	organic hydroperoxide resistance protein	YP_223139.1	Q579A6	140	BruAb2_0347	8	14337	14232	5.63	13	unknown	R: General function prediction only

^a^Gene ID, protein ID, accession no. and locus tag were retrieved from the NCBInr database.

^b^Theoretical molecular weight from the UniProtKB database entry.

^c^Subcellular locations were predicted using PSORTb v. 2.0.4.

^d^Cluster of orthologous groups (COG) protein database generated by comparing microbial genomes from the NCBI COG.

Multiple proteins that were immunoreactive at all stages of infection had varying p*I*, *M*_r_ and functions. The identified proteins were sorted into functional groups based on the classification of proteins encoded in complete genomes established by COG: 14 were related to transport and metabolism [4 for amino acids (spots 146, 178, 204, and 207), 3 to carbohydrates (spots 118, 164, and 231), 2 to inorganic ions (spots 239 and 240), 2 to nucleotides (spots 151 and 254), 2 to coenzymes (spots 161 and 169), and 1 to secondary metabolites (spot 218)]; 3 were involved in ribosomal structure and biogenesis related to protein translation (spots 162, 203, and 231), and 2 were associated with cellular processes and signaling, including post-translational modification, protein turnover, and chaperones (spots 228 and 253).

## Discussion

Brucellosis is a re-emerging zoonosis that has regained scientific attention because its pathogenesis in human and animal disease has significantly evolved [1,19]. However, the overall burden of this disease remains underestimated and is not well studied. The disease ecology has evolved rapidly in recent years, and there are novel populations with high risk of exposure and the potential to develop chronic or latent infection [20]. Eradication of brucellosis in animals is important for prevention of this disease in human beings and requires optimal diagnosis and vaccination [3]. There are relatively efficient diagnostic tests for brucellosis, and vaccines have been consistently developed; however, there are still several limitations [21,22]. Furthermore, cross-reacting bacteria decrease the specificity of the tests, and this has impeded the control of brucellosis [7]. To address these problems, it is important to develop new strategies for effective diagnosis with improved specificity. This study focused on identifying immunogenic proteins of *Brucella* from three different stages of infection (short-, middle-, and long-term) for the improvement of immunodiagnostics. Although distinct sets of *Brucella* antigens were only a limited set of proteins present at all three time points, these novel immunodominant proteins identified in our study might be suitable for the detection of *B. abortus* infection.

The 2DE gels containing *B. abortus* whole cell proteins were subjected to immunoblotting analysis using bovine antisera; the antisera were collected at three different phases of infection: the early (week 3), middle (week 7), and late (week 10) periods after challenge with *B. abortus*. An important diagnostic problem is the similarity of the O-antigenic side chains of *Brucella* and other Gram-negative bacteria such as *Y. enterocolitica* O:9 [7]. In this study, 25 protein spots reactive to *Y. enterocolitica* O:9-positive (YP) antisera and negative control (NC) sera in cattle were detected. By immunoblotting-linked gel image analysis of *B. abortus* proteins, the overlapping spots that were reactive to the YP and NC sera were excluded, and the spots that were reactive to the *B. abortus*-positive (BP) antisera at all stages of infection were selected; this analysis identified 120 distinct spots. The total number of spots reactive to the BP antisera at 3, 7, and 10 weeks post-challenge was comparable to the number of common spots (55) observed at all stages of infection. Furthermore, the common spots were 45.83% similar to those observed by immunoblotting using antisera from all three stages of infection; this suggests that the common immunoreactive spots might represent the proteins that are immunodominant at all stages of infection. The infection time-independent immunodominant proteins of *B. abortus* comprise proteins expressed from diverse genes encoding transport, metabolic functions and other immunogenic proteins. Previous studies have examined the immunogenicity of *Brucella* antigens but did not correlate this immunogenicity with the stage of infection. Therefore, this study identified several novel immunoreactive proteins in the bovine host based on the stage of infection.

Using the COG approach, most of the identified proteins were assigned functions related to the transport and metabolism of amino acids (60.9%). The 27.6 kDa DHDPR protein encoded by the *dapB* gene had the highest score value; DHDPR was proposed to function in the biosynthesis of lysine and diaminopimelate, but few proteomic studies have examined the role of this protein in *Brucella* infection [23]. The *dapB* in *Burkholderia pseudomallei* is an essential gene that was successfully mutagenized and identified as a beneficial marker [24]. Therefore, because the *B. abortus dapB*-encoded protein elicits an immunodominant response, this protein is a relevant candidate marker for infection. The predicted proteins involved in carbohydrate transport and metabolism included malate dehydrogenase (mdh), which functions in malate metabolism and the tricarboxylic acid cycle. mdh is expressed in response to acidic stress [25] and was broadly identified in *B. abortus* [10] and *B. melitensis* [26] using proteomic analyses. The second most frequent group included proteins related to ribosomal structure and protein translation. This group includes two ribosomal proteins, the 30S ribosomal protein S2 (RPS2) and the 50S ribosomal protein L25 (RPL25), and one translation elongation factor, EF-Ts (tsf). The RPS2 protein is highly conserved in prokaryotic-type ribosomes and is essential for binding of the ribosomal protein S1 to the 30S ribosomal subunit in *E. coli* [27]. In *Brucella*, RPS2 is repressed in response to oxidative stress [25] and is generally identified as the SSU ribosomal protein S1P of *B. melitensis* and *B. abortus* [10,26]. Additionally, elongation factor Ts (tsf), which is associated with protein translation, might be critical for the immunogenicity of *B. abortus*; this observation is consistent with previous data obtained by global protein analysis in *B. melitensis* [26]. Our study is the first to report that this protein is immunogenic at all stages of infection.

In the group of proteins involved in inorganic ion transport and metabolism, the 22.5 kDa Fe-Mn superoxide dismutase (Fe-Mn-SOD) detected at the p*I* of 5.58 is an oxidoreductase with superoxide dismutase activity. The Fe-Mn-SOD protein of *B. melitensis* is correlated with regulation of the stress response and was identified as a heat-shock protein (Hsp) [25]. Furthermore, the role of metal ions such as Fe and Mn in the response to relatively stringent environments has been elucidated with respect to *Brucella* pathogenesis [28]. Similar to the regulation of Fe- and/or Mn-SOD in response to heat shock and oxidative stress in some bacteria [29], *B. abortus* Fe-Mn-SOD is an essential factor that regulates specific stress responses inside hosts. Several molecular chaperones, including DnaK, GroEL and the HtrA protease, are known as stress proteins and virulence factors [25,30,31]; in our study, at least one chaperone protein, the pre-protein translocase subunit (SecB) was specific to a certain *B. abortus-*infection stage. SecB is a molecular chaperone specific to the proteobacteria, which comprises most gram-negative bacteria that are medically and industrially relevant [32]. SecB is required for the normal export of pre-proteins out of the cytoplasm, keeping them in a translocation-competent state.

Prevention of *Brucella* infections in livestock generally involves the use of live attenuated vaccines such as *B. abortus* (RB51 or S19) [33,34] and *B. melitensis* Rev1 [35]. S19 and Rev1 had the major disadvantage of inducing O-side chain-specific antibodies, which causes cross-reactivity during diagnosis; with RB51, the recovery of virulence was a major problem [36]. Consistently, several studies have focused on developing next-generation vaccines that are more safe and effective. Therefore, the immunogenic *Brucella* proteins identified in this study might provide supporting information for developing valid vaccine candidates that can elicit an efficient and specific immune response. Furthermore, it is important to consider the diagnostic method used depending on the animal and the stage of infection. Modern diagnostic methods are based on molecular approaches developed by proteomic analyses, and these advanced tools might soon replace the older, limited diagnostic methods. We suggest that the candidate proteins elucidated in this study might contribute a valuable solution to the present problems in the diagnosis of brucellosis, independent of the stage of infection. Ultimately, our investigation could provide helpful insight to advance the potential of immunogenic proteins as determinants for serological diagnosis and as novel tools for prevention of *Brucella* infection.
